# Nonfungible Tokens as a Blockchain Solution to Ethical Challenges for the Secondary Use of Biospecimens: Viewpoint

**DOI:** 10.2196/29905

**Published:** 2021-10-22

**Authors:** Marielle S Gross, Amelia J Hood, Robert C Miller Jr

**Affiliations:** 1 Department of Obstetrics, Gynecology and Reproductive Services University of Pittsburgh Medical Center Pittsburgh, PA United States; 2 Center for Bioethics and Health Law University of Pittsburgh Pittsburgh, PA United States; 3 Johns Hopkins Berman Institute of Bioethics Baltimore, MD United States; 4 Consensys Health Long Island City, NY United States

**Keywords:** blockchain, biospecimens, research ethics, nonfungible tokens, research ethics, health platforms, HeLa cells, patient data, deidentification, eHealth, data security, integrity

## Abstract

Henrietta Lacks’ deidentified tissue became HeLa cells (the paradigmatic learning health platform). In this article, we discuss separating research on Ms Lacks’ tissue from obligations to promote respect, beneficence, and justice for her as a patient. This case illuminates ethical challenges for the secondary use of biospecimens, which persist in contemporary learning health systems. Deidentification and broad consent seek to maximize the benefits of learning from care by minimizing burdens on patients, but these strategies are insufficient for privacy, transparency, and engagement. The resulting supply chain for human cellular and tissue–based products may therefore recapitulate the harms experienced by the Lacks family. We introduce the potential for blockchain technology to build unprecedented transparency, engagement, and accountability into learning health system architecture without requiring deidentification. The ability of nonfungible tokens to maintain the provenance of inherently unique digital assets may optimize utility, value, and respect for patients who contribute tissue and other clinical data for research. We consider the potential benefits and survey major technical, ethical, socioeconomic, and legal challenges for the successful implementation of the proposed solutions. The potential for nonfungible tokens to promote efficiency, effectiveness, and justice in learning health systems demands further exploration.

## Introduction

Deidentifying biospecimens “checks the box” of protecting privacy while permitting unrestricted secondary use of clinical data. This workaround transformed Henrietta Lacks’ [1] cervical cancer into death-defying HeLa cells (the paradigmatic learning health platform). According to convention in the then-segregated 1951 Johns Hopkins Hospital, tissue obtained during Henrietta Lacks’ cancer treatment was deidentified using the first 2 letters of her first and last names, permitting research on her tissue without her explicit knowledge or consent. Ms Lacks died soon after the procedure that harvested the would-be HeLa cells, leaving her family to mourn their loved one without realizing how the cancer that took her life also enabled her to live on through ongoing replication, distribution, and use of her tissue in perpetuity.

Open access to HeLa cells allowed scientists to ask and answer questions about a wide range of viruses, toxins, drugs, and hormones, while avoiding physical risk for human subjects, accelerating a revolution in biomedical science. Decades later, the provenance of the now ubiquitous HeLa cells came to light [2], when chance reidentification and subsequent efforts to obtain more samples from her living relatives exposed deficiencies in transparency, accountability, and engagement [3] throughout the learning health lifecycle. Today, Henrietta Lacks is recognized as the mother of modern medicine, the bittersweet result of a system that seeks to maximize the research value of tissue and other data-rich byproducts of clinical care.

Though our technical standards for deidentification have evolved, the spirit of deidentification that disconnected Ms Lacks, a poor, Black, mother of 5, from her legacy remains immortalized in US law and is widely exploited by today’s research enterprise. We discuss how deidentification potentiated ethical violations in Ms Lacks’ case, drawing parallels to contemporary research practices, and propose that nonfungible tokens (NFTs), an innovation building on blockchain technology, may help create a more ethical system for learning from care. The vastness of digital and genomic data has rendered all matter of biospecimens similarly undeidentifiable [4,5]. This use case focuses on excess surgical biospecimens and derived human cellular and tissue–based products; however, similar arguments may apply to digital data and related proprietary algorithms.

## Ethical/Legal Context for US Biomedical Research and Clinical Care

Physicians’ fiduciary duty to maintain patient confidentiality is enforced by the Health Insurance Portability and Accountability Act (HIPAA) Privacy Rule (45 CFR 164). The Belmont Report–inspired [6] Common Rule (45 CFR 46) stipulates that respect for persons and risk of “therapeutic misconception” require research participation to be transparent and voluntary, typically fulfilled by prospective informed consent. In this context, the “safe harbor” for deidentified data is justified by assumptions that privacy is patients’ only relevant interest regarding secondary research on the tissue and other data produced during clinical care and that deidentification offers sufficient privacy protection. Thus, preemptive deidentification establishes exemptions from HIPAA and human subject research protections.

Current learning platforms leverage deidentified biospecimens to accelerate scientific breakthroughs, empowering precision medicine [7]. Traditional expectations of research participation as altruistic and primarily beneficial for *other future patients* had implications for the structure of benefit distribution in learning health systems. US law prohibiting reidentification and contact (45 CFR § 164.514) prevents individuals from receiving timely access to relevant benefits that may be produced from research on their data. This is especially problematic for patient-centered outcomes research, which focuses on outcomes that matter to patients, and precision medicine research, which seeks to learn from and improve clinical care in real time [8,9].

Patients’ tissue and sensitive data are extracted during clinical care under fiduciary duty to benefit them and minimum necessary standards of Protected Health Information (PHI) (45 CFR 164.502(b), 164.514(d)). Systematic deidentification and repurposing of biospecimens without either explicit permission for research use or an intention to directly benefit that individual wherever possible may betray the sacred trust of the patient-physician relationship, compromising respect for patients as persons. Lack of transparency regarding relevant outcomes may contradict obligations of beneficence and information unblocking mandates set forth in the 21st Century Cures Act [10]. Direct, continuous, and ongoing research feedback is feasible with current bioinformatic technologies and programmable terms of use. Allowing indefinite delays in translation from the bench or the cloud back to the bedside of origin represents a lack of accountability to patients, which disproportionately impacts underserved populations who experience the greatest barriers for accessing cutting-edge care [11].

## Hidden in Plain Sight: Deidentification as Erasure Without Engagement

Henrietta Lacks was eventually reidentified, though she was never truly deidentified. Indeed, the Lacks family was easy to find when their help was needed to clear up HeLa contamination in the 70s [2]. The ability to identify a biospecimen’s source remains central for the integrity and value of related research. Likewise, current deidentification techniques are a similarly thin veneer of privacy protection [4,5]. Biospecimens are inherently unique; truly deidentifying them may not be possible given the richness of underlying data, advances in genomics, and maturation of artificial intelligence technology [4,5,12].

Unlike methods of concealing or encrypting personal identity and other data security measures, removing personal details impoverishes data sets, reinforces knowledge silos, and hinders continuous global assessment of the data landscape [13-15]. In addition to compromises on identity protection and scientific progress, deidentification prevents individuals from dynamically controlling or directly benefiting from the use of their biospecimens or understanding how they may have benefited others. Ms Lacks did not live to see the transformative effect she had on the world, but she had access to state-of-the-art care and the physician scientist who granted her cells immortality informed her of their world-changing potential. She “was glad her pain would come to some good for someone” [1].

Further harms experienced by obtaining and distributing the Lacks’ health data (ie, harvesting her tissue postmortem, seeking DNA from descendants, and publishing her genome, all without adequate transparency or consent) represented lost opportunities for recognition spanning decades, keeping wounds fresh and distributing their effects. The Lacks family narrative demonstrates an intergenerational harm of erasure that compounds the suffering and loss of illness. Subsequent engagement of the Lacks family on a National Institutes of Health review board overseeing HeLa cell use in government-funded research [16] represents a structural breakthrough in transparency, accountability, and engagement. This approach acknowledges the intergenerational nature of tissue and related learning, but has not yet been extended to the families of other patients whose biospecimens have generated foundational learning health platforms.

Like HeLa cells, our biospecimens are the substrate of learning health platforms, and are similarly deserving of respect. Current paradigms use deidentification to mitigate tradeoffs between privacy and utility of health data, allowing rapid scaling of learning platforms in tandem with digitalization. However, the resulting system effectively imposes the violations Ms Lacks experienced on all patients. How this resource-intensive model of engagement scales is unclear and complicated by uncertainty regarding future utility, the differential value of patients’ contributions, and the diversity of preferences [17]. Irrespective of whether the Lacks’ current engagement model should be normative rather than exceptional, further technological advancements may be essential for a learning health system that optimizes individual and collective rights and interests.

## Technological Solutions With Blockchain Technology and NFTs

As we strive to fully integrate care and research, advancements in blockchain technology and related privacy and intellectual property–preserving innovations may help embed ethical principles in learning health system architecture [18]. The decentralization of blockchains provides uniquely strong assurances of trust in data security, integrity, and use as the network is surveilled and audited by autonomous “smart contracts.” The fundamental transparency of the blockchain could enable individuals to track biospecimen use, and smart contracts could automate translation of potential health or personal benefits. Auditability of the learning health system may be crucial for ensuring that past, present, and future uses of human tissue and other clinically derived data are consistent with communal values.

Blockchains are communities of stakeholders organized around interoperable open-source building blocks with shared standards and information, in which sharing is normative, incentivized, and yields collective benefits. Blockchains’ underlying ethos of peer-to-peer engagement and cooperation could serve as the backbone of a learning health system that is designed to engage patients as proper stakeholders in learning, like the Lacks’ current oversight of HeLa cell use. Such a system could drive learning and translation by asserting patients’ values as contributors and empowering enforceable dynamic consent. Democratic engagement in system governance could dictate learning priorities, informed consent requirements appropriate to specific data use contexts, and operational aspects of participation. Importantly, advanced cryptography of blockchain networks allows transparent public engagement with individuals without compromising private identity.

Blockchains are often accompanied with their own cryptocurrency (tokens) to incentivize disparate parties to organize around a common purpose. Tokenization could incentivize patients to contribute their excess tissue to learning activities by providing transparency, trust, and feedback with a durable digital asset that may accrue in personal and health value over time. This supports the imperative to legitimize and democratize citizen science [19] and could provide suffrage and collective representation for patient advocacy movements [20]. Smart contract infrastructure may enable dynamic patient engagement for specific commercial tissue uses. Tokenization may facilitate development of a system for fairly compensating and maintaining transparency with individuals whose biospecimens become the basis of commercial tissue–based products [21].

NFTs, popularized by application to digital artworks [22], introduce the capacity to protect patient rights and interests regarding use of their inherently unique biospecimens and immortal cell lines by creating a corresponding unique digital asset that retains value even as the products are copied and distributed. NFTs could enable exchange of biospecimens to maximize research utility while retaining the unique signature of their human source. NFTs may allow us to capture and prove the value underlying health data without compromising individual or collective benefits of learning from biospecimens or privacy interests. This presents an opportunity for a paradigm shift in the recognition of nonfungible human beings as the basis of learning health platforms with a potential mechanism for ensuring a more just distribution of benefits. NFTs should be explored for their potential to empower provenance of data and duty in a system of learning from care.

Unsolved technical challenges remain for implementing and scaling NFTs for biospecimens. However, increasing adoption of decentralized ledger technologies in health care and beyond, as well as recent successful scaling operations utilizing privacy-preserving technologies (eg, federated learning [23] and homomorphic encryption [24]), support this further development of an ethical learning health system. Holistically, these innovations are ethically significant for learning health systems given their potential to resolve tensions between data utility and privacy; however, a more detailed discussion is beyond the scope of this article. Blockchain has since evolved to incorporate novel architectures to promote equitable collaborations (eg, Decentralized Autonomous Organizations [DAO]) and scalability of resource-intensive decentralized consensus mechanisms (eg, proof-of-stake algorithms). In the context of research on biospecimens, the use of these technologies must also be accompanied by examination of underlying ethical, legal, and social structures.

## Ethical and Socioeconomic Constraints of Tokenizing Human Tissue

The moral vulnerabilities, legal limitations, and practical constraints of tokenizing biospecimens are significant. Tokenization of tumors could incentivize inappropriate health risks on the part of stakeholders, including patients, physicians, and health systems. Secondary gain could motivate patients to delay surgery to allow for larger more valuable tumor samples, and physicians may be pressured to be less thorough in clinical pathologic examinations or more extensive in surgical interventions to maximize tissue yield. An ecosystem of human tissue tokens must anticipate and guard against potential abuses, including those related to monetization, and further tokenomic research can inform the optimal market design. Blockchain may enhance ethical protections for patients and subjects via an embedded approach to ethical oversight that is continuous, evolving, decentralized, and auditable.

In some settings, return of results raises concerns about the potential harms of disclosing information that may either be unwanted or have clinical consequences, including psychological or physical sequelae of subsequent interventions. While these challenges must be addressed, they do not justify acceptance of the status quo in which patients remain disconnected from research results that may be clinically actionable. NFTs could facilitate incorporation of dynamic consent, allowing patient preferences to guide tissue use and benefit distribution. Additional work is needed to determine appropriate informed consent and engagement of diverse populations. Further innovations and clinical pathways must be advanced to ensure safety, quality, and consent for returning results and delivering health benefits of biospecimen research.

The impact of tokenization on patients’ willingness to contribute biospecimens must be further evaluated, as some patients have expressed a preference for no-strings-attached donations, although this perspective may not account for the potential for that individual to receive substantial health, personal, or financial benefits. Tools are needed to socialize the outlook that patients can, and should, directly benefit from research on their tissue, and to communicate the potential for blockchain technology to resolve ethical and technical barriers. Novel economic structures, such as curated markets [25], augmented bonding curves [26], and platform cooperatives [27], could be employed in combination with underlying technical structures to optimally align stakeholder incentives, and research into these methods is ongoing. If tokenization leads to monetization of biospecimens, market design must optimize the use of rare uniquely valuable samples and ensure equitable distribution of benefits, and will require the support of updated legal protections.

## Legal and Practical Barriers for an NFT-Based Learning Health System

Aside from how NFTs could work for prospective biospecimens, retroactive application may be challenged by US regulations prohibiting reidentification and contact of deidentified research subjects. Since reconnecting patients with the knowledge and products from past biospecimen use is a major value proposition, devising strategies that do not violate established legal and ethical obligations will be critical. NFTs may circumvent prohibitions against reidentification, regardless of whether broad consent was initially obtained or not mandated for past specimen uses for which there was no expectation of contact, as they could provide patients an opportunity to opt in for subsequent reidentification, while providing discretionary preferences that could be updated over time. This overcomes the patient-facing concerns about privacy and autonomy, but does not eliminate institutional and researcher concerns about obligations or past lapses in disclosure that may arise.

By comparison with US law, the General Data Protection Regulation (GDPR) seeks to center individuals’ data rights as a matter of liberal democracy. A major challenge for the GDPR is the lack of mechanisms for enforcing ethical principles and socioeconomic policies for data use. Despite advantages for consumer autonomy, the GDPR has also been challenged as its limitations on secondary data use may frustrate efforts to maximize the individual and collective value of learning from health data [28]. Endless cookie requests signal consent but may not constitute meaningful control, especially if acceptance of cookies predicates access to needed health services. In practice, GDPR requirements resemble broad consent for the secondary use of biospecimens, aligned with the updated Common Rule [29]. Critically, the GDPR does not address the duty to distribute knowledge and products derived from secondary data and tissue use. Without a means of ensuring a just distribution of benefits, the underlying power asymmetry between individuals and third party data users persists.

Acceptability for current institutional tissue “owners” will be a major barrier to the implementation of NFTs for biospecimens, as fears of adverse press, decreased contributions, and legal repercussions from efforts to tokenize tissue loom large. “Safe harbor” for deidentified data and unsuccessful prior attempts of patients to obtain rights regarding the commercial use of their biosamples may reinforce institutional inertia [30]. Using NFTs for biospecimens recognizes their status as assets and may increase the call for updating definitions regarding rights, ownership, and value distribution.

Maximizing efficiency, effectiveness, and justice will ultimately require global collaboration in learning from care. Novel approaches to data governance must go above and beyond existing policy protections with an eye toward technological evolution and international standards. Transparency, auditability, and smart contract architecture could empower individuals or their representatives to ensure that data use is in accordance with policies and preferences while maximizing collective benefits. Additional measures for enforcing legal compliance, social democratic governance, and ethical oversight are needed to guard against potentially exploitative treatment of vulnerable populations within high income settings and worldwide.

## Conclusion

Henrietta Lacks’ story highlights the harms that may occur when deidentification separates research on patient tissue from obligations to promote respect, beneficence, and justice for that individual. Continued reliance on deidentification and broad consent for the “secondary use” of biospecimens may create platforms for learning that recapitulate historically exploitative practices of integrating research and patient care. Blockchain technology promises to build unprecedented transparency, engagement, and accountability into learning health system architecture. NFTs have the potential to embed the primacy of clinical ethics into our clinical research supply chains. HeLa cells are the original “use case” for NFTs, as they demonstrate the imperative of maintaining the provenance of nonfungible human-derived assets and the fiduciary duties to respective patients. Representing biospecimens with NFTs may maximize efficiency, effectiveness, and justice in the future of learning health systems.

## Abbreviations

GDPR: General Data Protection Regulation
HIPAA: Health Insurance Portability and Accountability Act
NFT: nonfungible token
